# Integrating Pharmacology and Gut Microbiota Analysis to Explore the Mechanism of Citri Reticulatae Pericarpium Against Reserpine-Induced Spleen Deficiency in Rats

**DOI:** 10.3389/fphar.2020.586350

**Published:** 2020-10-20

**Authors:** Yuying Zheng, Xuan Zeng, Pan Chen, Tingting Chen, Wei Peng, Weiwei Su

**Affiliations:** Guangdong Engineering and Technology Research Center for Quality and Efficacy Re-evaluation of Post-Market Traditional Chinese Medicine, Guangdong Provincial Key Laboratory of Plant Resources, School of Life Sciences, Sun Yat-sen University, Guangzhou, China

**Keywords:** Citri Reticulatae Pericarpium, spleen deficiency, gut microbiota, metabolites, network pharmacology

## Abstract

Citri Reticulatae Pericarpium (CRP), dried peels of *Citrus reticulata* Blanco and its cultivars, is an important traditional Chinese medicine for the treatment of spleen deficiency-related diseases. To date, the mechanism of CRP alleviating spleen deficiency has not been well investigated. This study aimed to explore corresponding mechanisms with integrating pharmacology and gut microbiota analysis. Firstly, the therapeutic effects of CRP against spleen deficiency were evaluated in reserpine-treated rats. CRP was found to effectively relieve the typical symptoms of spleen deficiency, including poor digestion and absorption capacity, and disorder in gastrointestinal hormones, immune cytokines and oxidative stress. Secondly, high throughput 16S rRNA gene sequencing revealed that CRP could not only up-regulate some short-chain fatty acids producing and anti-inflammatory bacteria but also down-regulate certain spleen deficiency aggravated related bacteria, eventually led to the rebalance of gut microbiota in spleen deficiency rats. In addition, a total of 49 compounds derived from CRP were identified in rat urine using ultra-high performance liquid chromatography-quadrupole- time of flight tandem mass spectrometry. Network pharmacology analysis showed that apigenin, luteolin, naringenin, hesperidin, hesperetin, homoeriodictyol, dihydroxy-tetramethoxyflavone, and monohydroxy-tetramethoxyflavone were the core bioactive components for CRP against spleen deficiency. Further Gene Ontology analysis and pathway enrichment suggested that therapeutic effects of CRP against spleen deficiency involved multiple pathways such as tumor necrosis factor signaling, hypoxia-inducible factor-1 signaling and Toll-like receptor signaling pathway. These results would help to understand the mechanism of CRP alleviating spleen deficiency and provide a reference for further studies.

## Introduction

In the theory of traditional Chinese medicine (TCM), spleen deficiency is a common clinical syndrome and described as symptoms such as emaciation, inappetence, epigastralgia, flatulence, lassitude, wilted complexion, loose stool, *etc*. Modern researches show that the spleen in TCM theory not only refers to the anatomical spleen, but also includes some functions of the pancreas and lymphatic system (Zheng et al., 2014). Spleen deficiency is a comprehensive manifestation of multiple functional declines, involving food digestion, nutrient absorption, energy metabolism, and immune system (Wang et al., 2016). Moreover, recent studies suggested that spleen deficiency is closely related to gut microbiota disorder (Qiu et al., 2017; Lin et al., 2018). Gut microbiota, as the main member of gut microecology, plays an essential role in the host’s health and can be deeply influenced by diets (Bibbò et al., 2016), antibiotics (Nogacka et al., 2018) and other environmental factors (Capurso and Lahner, 2017). In turn, the dysbiosis of the gut microbiota will act as a causative factor in gastrointestinal diseases (Feng et al., 2018). Recently, researchers found that some TCMs could exert their therapeutic effects on gastrointestinal diseases through regulating the balance of gut microbiota (Yang et al., 2017).

Citri Reticulatae Pericarpium (CRP), commonly referred to as Chenpi in Chinese, is derived from the pericarp of mature fruits of *Citrus reticulata* Blanco and its cultivars (State Pharmacopoeia Committee of People’s Republic of China, 2015). It is an important Chinese medicinal material which has been used for the treatment of respiratory and digestive diseases for thousands of years (Zheng et al., 2018). Long-term and extensive clinical applications have confirmed that CRP could alleviate multiple spleen deficiency related diseases, including indigestion, inappetence, abdominal fullness and distention (Yu et al., 2018). However, the mechanism of CRP alleviating spleen deficiency has not been well investigated. On the one hand, information concerning the bioactive components and potential targets involved in the effects of CRP against spleen deficiency are scarce. On the other hand, given the gut microbiota modulatory potential of CRP (Zeng et al., 2020a), it is meaningful to investigate the role of gut microbiota in the therapeutic efficacy of CRP.

In this study, reserpine-induced spleen deficiency rat model was established and used to evaluate the therapeutic effects of CRP. Reserpine, which was once used in the treatment of hypertension, could inhibit the vesicular monoamine transporter and deplete the brain monoamines such as 5-hydroxytryptamine by interfering with storage capacity, and ultimately lead to similar syndromes of spleen deficiency (Zhao et al., 2011; Maldonado and Maeyama, 2015). Meanwhile, spleen deficiency related disease targets were collected from accessible online databases and then used to explore the bioactive components and potential targets of CRP based on a network pharmacology strategy. Network pharmacology is a new discipline combined systems biology with drug efficacy, which helps to elucidate the inherent multi-component, multi-target, and multi-pathway characteristics of TCM (Ning et al., 2017).

Hereon, the therapeutic efficacy of CRP against spleen deficiency was evaluated in reserpine treated rats based on body signs and biochemical indexes including digestion, absorption, gastrointestinal hormones, immune regulation and oxidative stress. The gut microbiota modulatory properties of CRP in spleen deficiency rats were investigated with high throughput 16S rRNA gene sequencing. Moreover, CRP derived metabolites were identified using ultra-high performance liquid chromatography-quadrupole-time of flight tandem mass spectrometry (UHPLC-Q-TOF-MS/MS) and were further employed in network pharmacology analysis to capture the bioactive components and potential targets of CRP in treating spleen deficiency. This study would be helpful for further understanding of the pharmacological effects and therapeutic benefits of CRP against spleen deficiency related diseases.

## Materials and Methods

### Chemicals and Materials

CRP samples (Batch number: 201712) were acquired from Xinhui Hele Tea Art Co. Ltd. (Jiangmen, China), and were authenticated by Prof. Wenbo Liao from Sun Yat-sen University. Corresponding voucher specimens were kept in our laboratory. Reserpine injection was purchased from Guangdong Bangmin Pharmaceutical Co., Ltd. (Jiangmen, China). The reference standards of hesperidin, naringin, neohesperidin and rutin were obtained from National Institute for Control of Biological and Pharmaceutical Products of China (Beijing, China). Hesperetin, naringenin, nobiletin and mass spectrometry (MS) grade formic acid were purchased from Sigma-Aldrich (St. Louis, MO, United States). MS grade acetonitrile was purchased from Fisher Scientific (Pittsburgh, PA, United States). All water used was distilled and further purified by a Milli-Q system (Millipore, Milford, MA, United States). Other reagents used in the experiment were of analytical grade.

### Preparation of Citri Reticulatae Pericarpium Extract

The CRP sample (20 g) was cut into small pieces, and soaked in boiled distilled water for three times (2, 1.5 and 1.5 L of each bulk, respectively; 20, 15 and 15 min of each time, respectively). After filtration, the whole extracts were evaporated to 500 ml with a rotary evaporator (Eyela, Tokyo, Japan) at 60°C, to obtain the CRP extract with a concentration of 0.04 g mL^−1^.

### Animals and Experimental Design

Thirty male Sprague-Dawley rats (weighting 180–220 g) were purchased from Guangdong Medical Laboratory Animal Center, and maintained in standard temperature conditions (20–23°C) and a 12/12-h light-dark cycle, with food and water supplied *ad libitum*. All experimental procedures were approved by the Animal Ethics Committee of the School of Life Sciences in Sun Yat-sen University and carried out in accordance with the National Institutes of Health guide for the care and use of Laboratory animals (NIH Publications No. 8023, revised 1978).

Rats were randomly divided into three groups (control group, model group and CRP group). Rats in model and CRP group were injected subcutaneously with reserpine injection at 0.5 mg kg^−1^ d^−1^ for 14 days to induce spleen deficiency (Wang et al., 2016). Rats in the control group were injected with the same volume of saline. After that, rats in CRP group were intragastrically given CRP extract (0.04 g ml^−1^, 15 ml kg^−1^ d^−1^) for 14 days. Rats in control and model groups received distilled water.

After the last administration, rats were housed individually in metabolic cages (Y-3102, Yuyan Instruments Co. Ltd.; Shanghai, China), with fasting and free access to water. Urine samples were collected within 12 h post-dose for metabolite identification. Subsequently, about 1 ml blood was sampled from retro orbital plexus, and then 5 ml 3% D-xylose solution was intragastrically administered to rats. Exactly 1 h later, rats were anesthetized with 10% chloral hydrate (3 ml kg^−1^) to collect blood samples from abdominal artery. Serum was obtained after centrifugation. In addition, feces samples for gut microbiota analysis were collected from the rectum, transferred into sterile conical tubes, and then immediately frozen in liquid nitrogen. Obtained samples were stored at −80°C until analysis.

### Kits Tests

The activities of superoxide dismutase (SOD) and amylase in serum, as well as the concentrations of D-xylose and malondialdehyde, were determined following protocols of corresponding Kits (Nanjing Jiancheng Bioengineering Institute, Nanjing, China). The concentrations of gastrin, motilin, cholecystokinin-8 (CCK-8), interleukin 6 (IL-6) and tumor necrosis factor-alpha (TNF-α) in serum were detected using commercial ELISA kits (Uscnlife Sciences & Technology Co., Wuhan, China) according to the manufacturer’s instructions.

### Gut Microbiota Analysis Using 16S rRNA Gene Sequencing

Total bacterial DNA were extracted and amplified as previously described (Zheng et al., 2020). After PCR amplification, sequencing was performed on an Illumina Hiseq 2500 platform by Biomarker Technologies Co. Ltd. (Beijing, China). Bioinformatics analysis was performed based on operational taxonomic units, which were clustered based on a 97% sequence similarity according to UCLUST (Edgar, 2010). For alpha diversity analysis, Chao1, ACE, Shannon index, and Simpson index were calculated in the QIIME program (version 1.8) (Caporaso et al., 2010). For beta diversity analysis, principal coordinate analysis (PCoA) was performed under the Gower algorithm. All processes were performed on the BMKCloud platform (www.biocloud.net). The Spearman’s rho non-parametric correlations between gut microbiota (the top 60 genera in relative abundance) and spleen deficiency related indexes were then calculated by using SPSS software (version 22.0) and displayed by R software with a pheatmap package.

### Identification of Metabolites Derived From Citri Reticulatae Pericarpium

To identify CRP derived metabolites, an aliquot of 100 μL urine sample was vortex-mixed with 200 μL volume acetonitrile for 3 min, and centrifuged at 15,000 × g for 30 min at 25°C. Finally, 10 μL supernatant was subject to UHPLC-Q-TOF-MS/MS analysis.

Detection of CRP derived metabolites was carried out using an ultra-fast liquid chromatography (Shimadzu Corp., Kyoto, Japan) coupled with quadrupole/time-of-flight mass spectrometry (Triple TOF 5600 plus, AB SCIEX, Foster City, United States). Gradient chromatographic separation was performed on a Kinetex C_18_ column (2.6 μM, 150 mm × 3.0 mm) and maintained at 40°C. The mobile phase consisted of acetonitrile (A) and water containing 0.1% aqueous formic acid (*v/v*) (B). The elution was carried out following the program: 5–100% A (0–30 min), 100% A (30–34 min) with the flow rate kept at 0.3 ml/min. The mass spectrometry detector was equipped with an electrospray ionization source and operated under the same parameters with our reported studies (Zeng et al., 2019).

Data acquisition was carried out using Analyst® TF 1.6 software (AB Sciex, Foster City, United States) in information-dependent acquisition mode. Metabolite identification was based on chromatographic elution time, chemical composition, MS/MS fragmentation pattern, and comparisons with available standards and references.

### Network Pharmacology Analysis

#### Collection of Potential Targets for Citri Reticulatae Pericarpium

With the UHPLC-Q-TOF-MS/MS system, a total of 49 metabolites were characterized in rat urine. The associated proteins of CRP were searched in Comparative Toxicogenomics Database (Davis et al., 2019), Encyclopedia of TCM (Xu et al., 2019) and Swiss Target Prediction Database (Gfeller et al., 2014).

#### Collection of Potential Targets for Spleen Deficiency

As there are no therapeutic targets about spleen deficiency that were available directly in the databases, targets of diseases that have similar pathological features with spleen deficiency were collected as alternatives. Diarrhea, dyspepsia, enteritis and gastritis were chosen out, and the corresponding targets were searched in DisGeNET (Pinero et al., 2020).

#### Protein-Protein Interaction Analysis

Protein-protein interaction (PPI) data was constructed by inputting official gene symbol to the “Multiple Proteins” search on String website (Szklarczyk et al., 2019), with organism species limited to “Homo sapiens” and a confidence score >0.9. The interaction results were exported as (.tsv) file for further network analysis by using Cytoscape software. The CRP-related target-spleen deficiency target network (CT-ST network) was constructed based on their PPI data and visualized by Cytoscape 3.7.0 software. The CRP-related targets were mapped to the spleen deficiency-related targets for obtaining the common targets of both. Then a network including the common targets and their first neighbors was extracted from the CT-ST network. In the generated networks, nodes represent targets and components, and edges represent the relationship between them. The targets without interaction were excluded from the network. Afterward, a plugin of Cytoscape (Network Analyzer) was applied to analyze the topological parameters of each node in the network. Among the topological parameters, degree, betweenness centrality and closeness centrality were used as crucial factors to evaluate the most influential nodes in networks. The nodes with “Degree” value greater than twofold the median value of all the network nodes, “Betweenness centrality” and “Closeness centrality” value greater than the median value of all the network nodes were chosen as the key targets.

#### Gene Ontology Enrichment and Pathway Analysis

The Database for Annotation, Visualization and Integrated Discovery was applied for Gene Ontology (GO) enrichment and pathway analysis (Jiao et al., 2012). The specific operation steps were as following, inputting the protein ID and restricting the species to “Homo sapiens,” then utilizing the functional annotation tool to make GO enrichment and pathway analysis.

### Statistical Analysis

Data were expressed as mean ± standard deviation (*n* = 10). The significant differences between the groups were assessed by ANOVA test in SPSS software (version 22.0), and *p* values less than 0.05 or 0.01 were considered as significant difference. Figures were plotted with GraphPad Prism (Version 7.00).

## Results and Discussion

### Therapeutic Effects of Citri Reticulatae Pericarpium Against Spleen Deficiency

**FIGURE 1:**
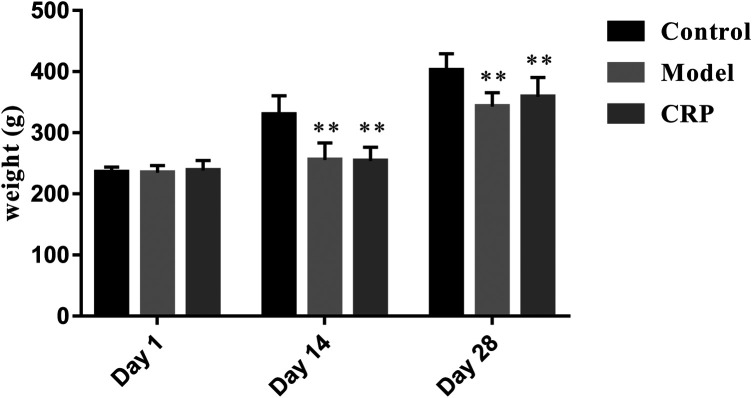
Rat body weights comparison between control, model and CRP treated group. (*n* = 10, **p < 0.01, vs. control group. Although the body weights of CRP treated group were higher than that of model group after intervention, there existed no significant difference.)

TCM clinical syndromes are often difficult to quantify with modern analysis methods. How to use animal models to characterize the syndromes is one of the difficulties in the modern researches on TCM (Meng et al., 2013). In this work, a reserpine-induced spleen deficiency rat model was established to evaluate the therapeutic effects of CRP based on body symptoms and biochemical indexes. Three days after the subcutaneous injection of reserpine, rats showed typical symptoms of spleen deficiency, such as anorexia, weight loss, pasty loose stools, inactiveness, and grouping. As presented in Figure 1, reserpine treatment significantly reduced the body weight of rats (*p* < 0.01). With the administration of CRP for 14 days, above-mentioned symptoms of spleen deficiency in rats were significantly improved. Rat body weights of CRP group were higher than that of model group, although there was no significant difference. These results preliminarily suggested that CRP administration could relieve the symptoms of spleen deficiency induced by reserpine.

**FIGURE 2:**
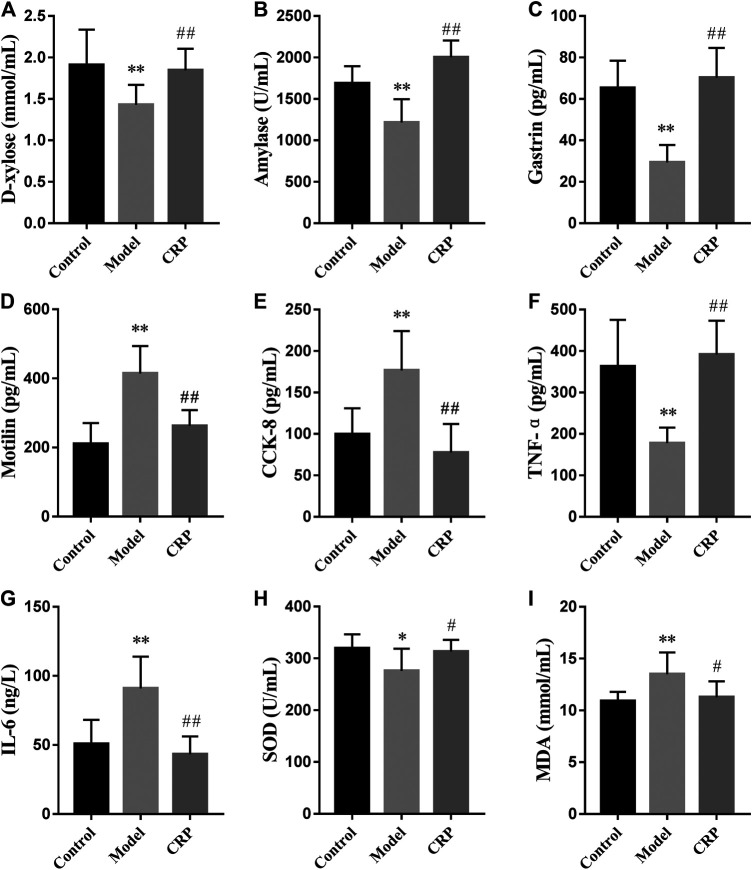
Effects of CRP on biochemical parameters in serum, including the concentrations of **(A)** D-xylose and **(I)** MDA, the activities of **(B)** amylase and **(H)** SOD, the levels of **(C)** gastrin, **(D)** motilin, **(E)** cholecystokinin-8, **(F)** TNF-α and **(G)** IL-6 (*n* = 10; ^*^ *p* < 0.05, vs. control group; **p < 0.01, vs. control group; ^#^ *p* < 0.05, vs. model group; ^##^ *p* < 0.01, vs. model group).

Subsequently, we further investigated the effects of CRP on biochemical indexes in reserpine-induced spleen deficiency rats, including the parameters used to characterize digestion, absorption, gastrointestinal hormones, immune regulation and oxidative stress. Obtained results were illustrated in Figure 2. Reported studies have observed that patients with spleen deficiency usually showed the symptoms of poor digestion and absorption (Chung et al., 2016). In this study, the activity of amylase and the concentration of D-xylose in serum were used to reflect the digestion and absorption of nutrients in rats (Gao et al., 2009; Li et al., 2020). As shown in Figure 2, the level of D-xylose and the activity of amylase in model group were significantly lower than that in control and CRP group (*p* < 0.01), suggesting that reserpine treatment could significantly reduce the digestion and absorption in rats while CRP administration could effectively reverse these changes.

Gastrointestinal hormones take an important role in regulating secretory and motor functions of the digestive tract (Xie et al., 2006). It has been found that the levels of gastrointestinal hormones in patients with gastrointestinal diseases were different from those in normal individuals (Wang et al., 2013). Hereon, three representative gastrointestinal hormones (gastrin, motilin, CCK-8) were employed to evaluate the effects of CRP on neuroendocrine in spleen deficiency rats. Gastrin, released from the G cells in antrum and duodenum, could stimulate the secretion of gastric acid, pepsin, bile and improve gastrointestinal movement (Itoh et al., 2005). Motilin is a peptide synthesized by mucosal endocrine cells in the upper segment of small intestine. It could promote gastrointestinal motility and improve the contractility and tension of the gastrointestinal tract and biliary tract (Itoh et al., 2005). CCK-8, a peptide hormone widely distributed in gastrointestinal tract and brain, is experimentally identified as a transmitter involved in multiple physiological activities, such as acting on feeding center, causing satiety and inhibiting feeding (Stengel and Tache, 2011). As illustrated in Figure 2, compared with control group, the level of gastrin in model group significantly decreased while the levels of motilin and CCK-8 significantly increased (*p* < 0.01), showing that reserpine treatment led to the disorder of gastrointestinal hormones secretion in rats. Changes in these three gastrointestinal hormones caused by reserpine treatment were up to normal after the administration of CRP for 14 days, revealing the efficiency of CRP in improving digestive dysfunction through regulating neuroendocrine.

The dynamic balance between Th1 and Th2 immune response is important for the immune system (Bashyam, 2007; Fischer et al., 2007). Th1 cytokines are with mediation in cell immunity response and mainly include IFN-γ, TNF-α, IL-1, IL-2, while Th2 cytokines mediate humoral immunity and include IL-4, IL-5, IL-6, IL-10 (Mosmann et al., 1986). In the model group, reserpine treatment significantly decreased the level of TNF-α while promoted that of IL-6 in serum (*p* < 0.01). After the intervention with CRP, the level of TNF-α and IL-6 restored to normal, suggesting that CRP could help maintain the balance of Th1 and Th2 immune response.

In addition, the balance between oxidants and antioxidants is necessary in maintaining health, and corresponding imbalance may result in oxidative stress causing functional disorders and certain diseases (Sharma et al., 2015). In this study, rats in model group had lower activity of SOD and higher level of malondialdehyde (*p* < 0.05) than control group, indicating that reserpine treatment resulted in free radical disorder in spleen deficiency rats. CRP administration could improve the activity of antioxidant enzyme and reduce lipid peroxidation damage (*p* < 0.05).

In summary, CRP administration could effectively alleviate the syndromes of spleen deficiency induced by reserpine treatment in rats, including poor digestion and absorption capacity, and disorder in gastrointestinal hormones, immune cytokines and oxidative stress.

### Gut Microbiota Modulatory Effects of Citri Reticulatae Pericarpium in Spleen Deficiency Rats

**FIGURE 3:**
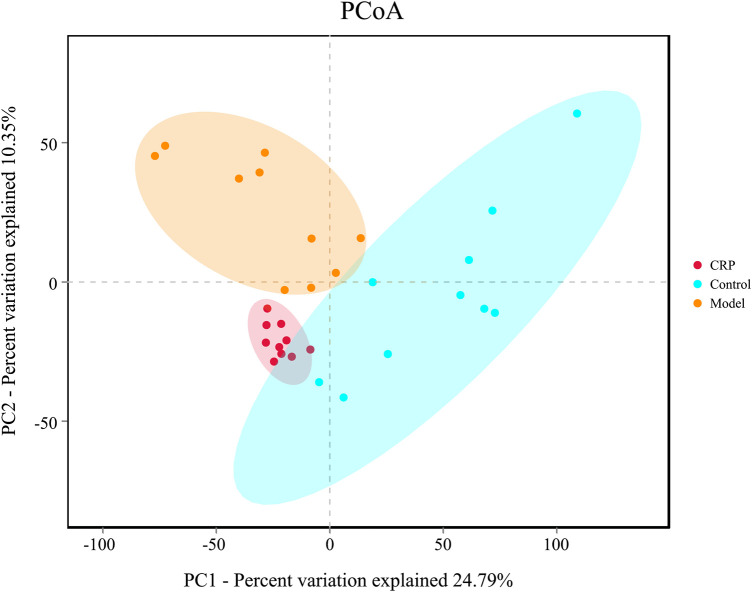
PCoA plot of microbial communities were based on OTU composition, each treatment group is represented by different color. *N* = 10 in each group.

Gut microbiota plays a vital role in maintaining normal intestinal functions, such as food digestion, nutrient absorption, integrity of epithelial barrier, and development of mucosal immunity (Veerappan et al., 2012). Recently, more and more evidence indicated that gut microbiota imbalance is a potential trigger for many diseases such as inflammatory bowel disease (Veerappan et al., 2012), diabetes (Meijnikman et al., 2018) and metabolic syndrome (Zeng et al., 2020a). In this study, the changes of bacterial richness (expressed in ACE and Chao1) and diversity (expressed in Shannon and Simpson index) in response to reserpine treatment and CRP intervention were investigated. As shown in Table 1, remarkably lower ACE, Chao1 and Shannon indexes were observed in the model group (*p* < 0.01), which suggested that reserpine treatment disturbed the balance of gut microbiota. By contrast, supplement with CRP gave rise to a higher community richness than that of the model group (*p* < 0.01), indicating that CRP is beneficial to the growth of gut microbiota. Beta diversity analysis among experimental groups was performed with PCoA. As shown in Figure 3, the PCoA score plot depicted three clearly divided groups: samples from the model group gathered in the second quadrant; samples from the control group dispersed in the first, third, and fourth quadrants; and samples from CRP group concentrated in the third quadrant, which were similar to that of control group. These results indicated that CRP intervention could help to restore changes in the richness and diversity of gut microbiota in spleen deficiency rats.

**TABLE 1:** The diversity index of gut microbiota in rats in different groups.

Group	Community richness		Community diversity	
	ACE	Chao1	Shannon	Simpson
Control	516.47 ± 24.71	524.54 ± 26.4	4.51 ± 0.23	0.03 ± 0.01
Model	396.41 ± 38.51^**^	407.74 ± 39.15^**^	4.06 ± 0.17^**^	0.04 ± 0.01
CRP	459.20 ± 37.60^##^	460.2 ± 34.76^##^	3.68 ± 0.18	0.07 ± 0.01

Note: *n* = 10, vs. Control: **p < 0.01; vs. Model: ^##^ *p* < 0.01.

**FIGURE 4:**
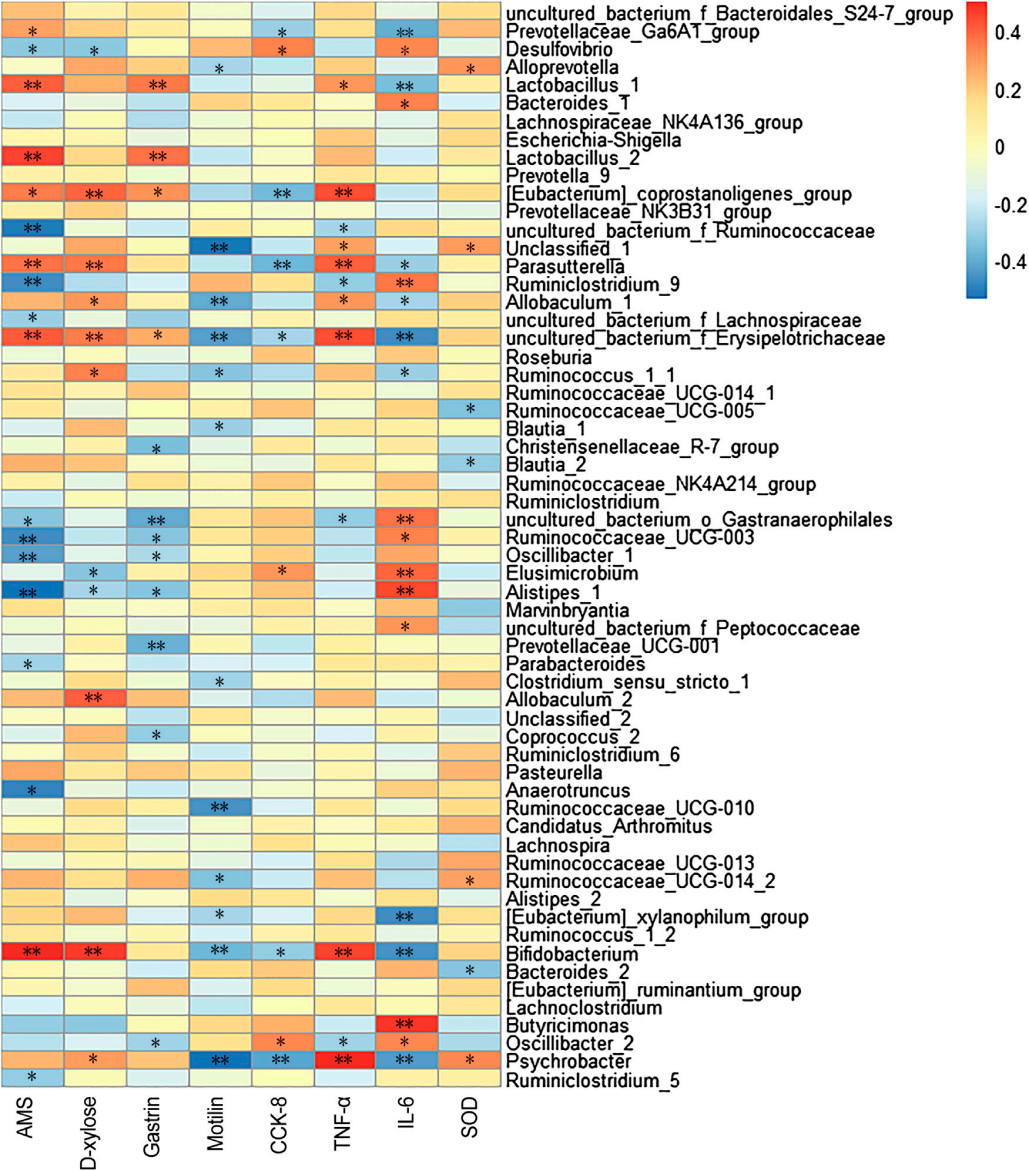
Heatmap of Spearman’s correlation between gut microbiota (the relative abundances of top 60 genus) and spleen deficiency related indexes. The colors range from blue (negative correlation) to red (positive correlation). Significant correlations are noted by ^*^ *p* < 0.05 and **p < 0.01.

The correlations between gut microbiota (the top 60 genera in relative abundance) and spleen deficiency-related indexes were calculated using Spearman’s rho non-parametric correlation analysis (Figure 4). The heatmap reflected significant positive correlations between the improvement of spleen deficiency related-indexes and some short-chain fatty acids (SCFAs) producing bacteria, such as *Bifidobacterium*, *Lactobacillus*, *Allobaculum*, *Psychrobacter*, *Prevotellaceae_Ga6A1_group*, *[Eubacterium]_coprostanoligenes_group*, *Parasutterella*, etc. SCFAs (acetate, propionate and butyrate), the end products of gut microbial fermentation of indigestible dietary components, appeared to enhance epithelial barrier function, improve gut permeability, inhibit the inflammation. Among them, *Bifidobacterium* and *Lactobacillus* are the most widely used probiotics with many health-promoting properties, such as prevention of enteropathogen colonization (barrier effects) (Candela et al., 2008), anti-inflammatory effects on mucosal surfaces, optimization of the composition of gut microbiota (O’Mahony et al., 2005). There has been growing interest in using probiotics as an adjunct to standard anti-inflammatory and immune suppressing therapy (Veerappan et al., 2012). *Allobaculum*, a butyrate-producing genus, has been reported to be an important functional phylotypes in many researches, its reduction was associated with obesity and diabetes (Zhang et al., 2015; Barouei et al., 2017). *Eubacterium coprostanoligenes group* can convert cholesterol to coprostanol which is poorly absorbed in human intestines and would be excreted, leading to blood cholesterol concentration reduction (Li et al., 1998) In addition, *Alloprevotella* was positively correlated with SOD activity, a genus that fermented carbohydrates and produced acetate and butyrate (Downes et al., 2013). Studies have illustrated that its abundance was negatively correlated with various diseases such as obesity, diabetes and cardiovascular diseases (Wei et al., 2018).

Moreover, significant negative correlations between spleen-deficiency aggravated related indexes and *Alistipes*, *Anaerotruncus*, *Desulfovibrio*, *Oscillibacter*, *Ruminiclostridium_9*, *Ruminococcaceae_UCG-003*, *Parabacteroides*, *Ruminiclostridium_5*, *etc.* Among them, *Desulfovibrio* is the predominant bacteria in human colon sulfate-reducing bacteria, which can reduce sulfate to produce H_2_S. Since endogenous H_2_S can poison intestinal epithelial cells, clinical studies have inferred that *Desulfovibrio* was associated with intestinal diseases (Gobert et al., 2016). It is reported that *Oscillibacter* was increased in the diet-induced metabolic dysfunctions, and associated with impaired intestinal barrier integrity (Lam et al., 2012).

**FIGURE 5:**
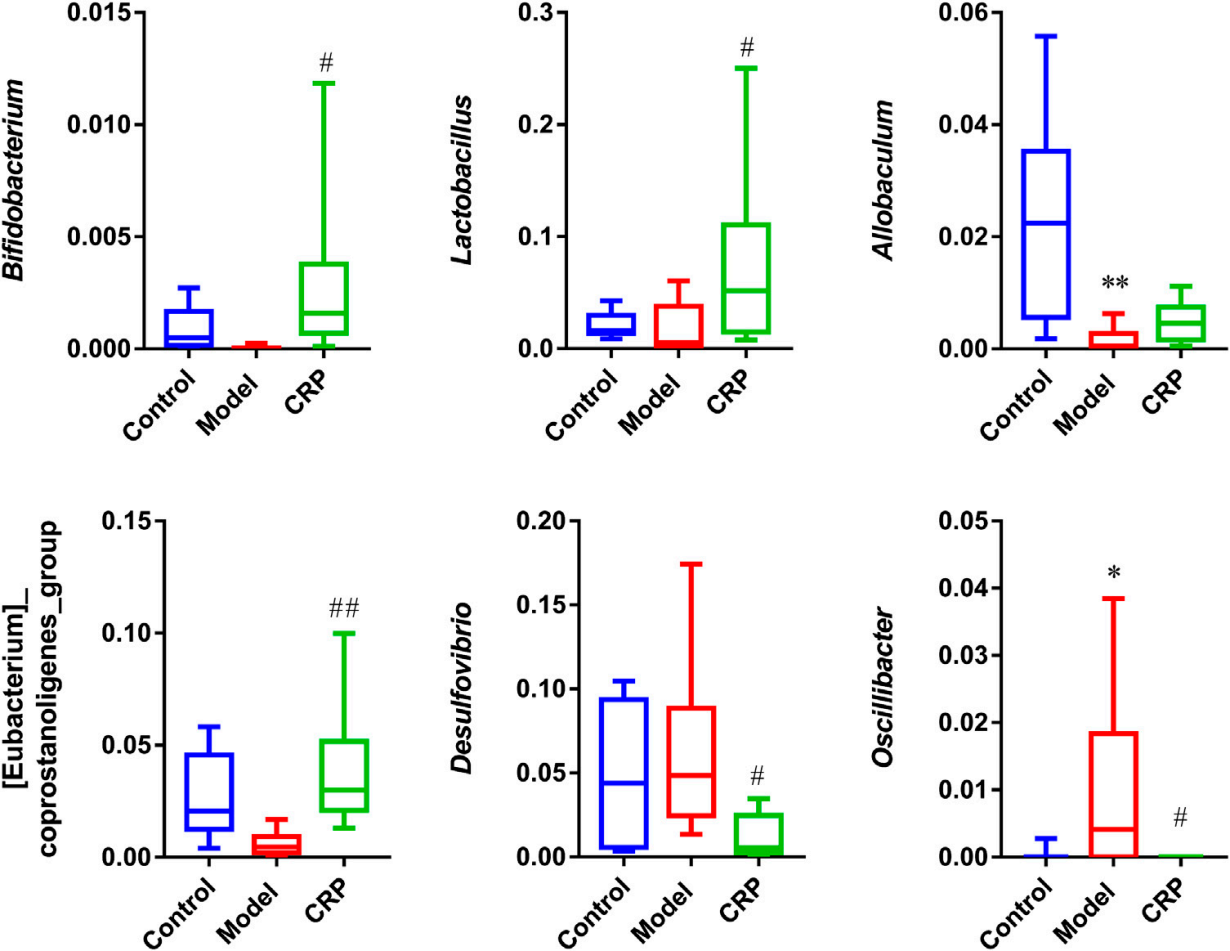
Relative abundances of *Bifidobacterium*, *Lactobacillus*, *Allobaculum*, *[Eubacterium]_coprostanoligenes_group*, *Desulfovibrio*, *Oscillibacter*. (*n* = 10; ^*^ *p* < 0.05, vs. control group; ^#^ *p* < 0.05, vs. model group; ^##^ *p* < 0.01, vs. model group.)

As described in our preliminary study, flavonoids are the primary components in CRP (Zheng et al., 2019). Flavonoids, as common dietary polyphenols, have been proven to exert potential modulatory effects on gut microbiota by inhibiting the growth of multiple pathogens and promoting beneficial genera. These modulations in turn promote gut health through maintaining gut immune homeostasis, and improving nutrients absorption (Dueñas et al., 2015; Pei et al., 2020). In this work, CRP exerted a significant effect on improving the composition of gut microbiota, especially for SCFAs producing and anti-inflammatory bacteria (Figure 5), whose metabolites could enhance epithelial barrier function, improve gut permeability, and inhibit inflammation. Based on these results, it is reasonable to propose that the relief of symptoms in spleen deficiency rats was closely associated with the rebalance of gut microbiota.

### Identification of Metabolites Derived From Citri Reticulatae Pericarpium

Generally, ingested exogenous compounds would undergo multi-step *in vivo* biotransformation processes mediated by gut microbiota and mammalian metabolic enzymes (Walle, 2004), which mainly comprise phase I and phase II metabolism. Phase I metabolism (hydrolysis, oxidation, demethylation, etc.) would change the skeleton structure of compounds, while phase II metabolism (glucuronidation, sulfation, acetylation, etc.) converts the compound into more water-soluble metabolites for excretion through urine (Gradolatto et al., 2004). Urine is filtered and concentrated from the blood through nephron. Metabolites in the blood are eventually excreted in the urine with higher concentrations, making them easier to be detected in urine by analytical instruments (Gao, 2015; Zhao et al., 2018). In addition, urine can be continuously sampled in a non-invasive way, and will not affect the body’s normal physiological processes. Hence, urine is probably a better source for metabolite identification than blood.

In this work, based on our preliminary results of chemical composition analysis (Zheng et al., 2019), a total of 26 prototype compounds and 23 metabolites were detected in rat urine after the ingestion of CRP. Prototype compounds were mainly polymethoxyflavones, as well as hesperetin, naringenin, isoprinol, luteolin and apigenin. Catalyzed by phase I and phase II metabolic enzymes, ingested prototype compounds underwent hydrolysis, demethylation, glucuronidation and sulfation, giving rise to a mass of metabolites. Compound descriptions, molecular formulas, retention times, and fragment ions of these prototype compounds and metabolites were detailed in Table 2.

**TABLE 2:** Identification of metabolites in rat urine, and feces samples after the oral administration of Citri Reticulatae Pericarpium extract.

Peak no.	Identification	Molecular formula	Retention time (min)	[M + H]^+^(*m/z*) (error, ppm)	[M − H]^−^(*m/z*) (error, ppm)	Fragment ions in the positive ion mode(*m/z*)^a^	Fragment ions in the negative ion mode(*m/z*)^a^
	Polymethoxyflavone derivates						
1	Monohydroxy-trimethoxyflavone	C_18_H_16_O_6_	14.7	329.1033 (2.2)	327.0879 (0.5)	314.0777 [M+ H-CH_3_]^+^, 299.0558 [M + H-2CH_3_]^+^, 285.0754 [M + H-CH_3_-HCO]^+^, 271.0600 [M + H-2CH_3_-CO]^+^, 243.0630, 229.0485, 181.0130, 153.0172	312.0688 [M − H-CH_3_]^−^, 297.0380 [M − H-2CH_3_]^−^, 282.2452 [M − H-3CH_3_]^−^, 177.0194
2	Monohydroxy-trimethoxyflavone	C_18_H_16_O_6_	15.7	329.1031 (2.7)	327.0885 (0.8)	313.0720, 299.0545 [M + H-2CH_3_]^+^, 285.0783 [M + H-CH_3_-HCO]^+^, 271.0609 [M + H-2CH_3_-CO]^+^, 268.0725 [M + H-2CH_3_-CH_3_O]^+^, 239.0696	312.0650 [M − H-CH_3_]^−^, 297.0430 [M − H-2CH_3_]^−^, 282.2433 [M − H-3CH_3_]^−^, 146.9376, 102.9446
3	Dihydroxy-trimethoxyflavone	C_18_H_16_O_7_	13.5	345.0987 (3.3)	343.0806 (1.6)	330.0712 [M + H-CH_3_]^+^, 315.0500 [M + H-2CH_3_]^+^, 287.0576 [M + H-2CH_3_-CO]^+^	328.0553 [M − H-CH_3_]^−^, 313.0407 [M − H-2CH_3_]^−^, 298.2388 [M − H-3CH_3_]^−^, 297.2190
4	Dihydroxy-trimethoxyflavone	C_18_H_16_O_7_	19.0	345.0963 (2.0)	343.0836 (0.7)	330.0748 [M + H-CH_3_]^+^, 315.0531 [M + H-2CH_3_]^+^, 284.0715 [M + H-2CH_3_-CH_3_O]^+^, 257.1077, 197.0121	328.05384 [M − H-CH_3_]^−^, 313.0351 [M − H-2CH_3_]^−^, 298.0067 [M − H-3CH_3_]^−^, 281.9727 [M − H-2CH_3_-CH_3_O]^−^, 255.9557, 208.0703, 166.0599
5	Trimethoxyflavone*-O-*glucuronide	C_24_H_24_O_12_	11.0	505.1350 (2.0)	503.1162 (−3.4)	329.1009 [M + H-GlcUA]^+^, 314.0760 [M + H-GlcUA-CH_3_]^+^, 299.0559 [M + H-GlcUA-2CH_3_]^+^, 249.0630	ND
6	Trimethoxyflavone*-O-*sulfate	C_18_H_16_O_9_S	12.6	409.0614 (2.6)	407.0434 (−2.1)	329.1041 [M + H-SO_3_]^+^, 314.0815 [M + H-SO_3_-CH_3_]^+^, 299.0563 [M + H-SO_3_-2CH_3_]^+^, 271.0593 [M + H-SO_3_-2CH_3_-CO]^+^, 243.0691, 181.0111	327.0869 [M − H-SO_3_]^−^, 312.0641 [M − H-SO_3_-CH_3_]^−^, 297.0427 [M − H-SO_3_-2CH_3_]^−^, 201.0307
7	Trimethoxyflavone*-O-*sulfate	C_18_H_16_O_9_S	13.8	409.0630 (5.8)	407.0477 (4.5)	329.1052 [M + H-SO_3_]^+^, 313.0761, 268.0755, 257.0416, 239.0620	327.0877 [M − H-SO_3_]^−^, 312.0682 [M − H-SO_3_-CH_3_]^−^, 297.0455 [M − H-SO_3_-2CH_3_]^−^
8	Trimethoxyflavone*-O-*sulfate	C_18_H_16_O_9_S	14.5	409.0721 (1.2)	407.0440 (−0.5)	329.1024 [M + H-SO_3_]^+^, 299.0566 [M + H-SO_3_-2CH_3_]^+^, 257.0455	ND
9	Monohydroxy-tetramethoxyflavone	C_19_H_18_O_7_	14.7	359.1133 (1.5)	357.0976 (−0.8)	344.0844 [M + H-CH_3_]^+^, 329.0648 [M + H-2CH_3_]^+^, 301.0720 [M + H-2CH_3_-CO]^+^, 285.0410	ND
10	Monohydroxy-tetramethoxyflavone	C_19_H_18_O_7_	15.0	359.1137 (3.5)	357.0972 (−1.3)	344.0869 [M + H-CH_3_]^+^, 329.0664 [M + H-2CH_3_]^+^, 314.0438 [M + H-3CH_3_]^+^, 301.0710 [M + H-2CH_3_-CO]^+^, 286.0485 [M + H-3CH_3_-CO]^+^, 181.0144, 153.0186	342.0704 [M − H-CH_3_]^−^, 327.0474 [M − H-2CH_3_]^−^, 312.0306 [M − H-3CH_3_]^−^, 269.0069
11	Monohydroxy-tetramethoxyflavone	C_19_H_18_O_7_	15.4	359.1111 (2.2)	ND	344.0889 [M + H-CH_3_]^+^, 326.0789 [M + H-CH_3_-H_2_O]^+^, 298.0848, 162.0690	ND
12	Monohydroxy-tetramethoxyflavone	C_19_H_18_O_7_	16.1	359.1136 (4.0)	357.0976 (−1.6)	344.0888 [M + H-CH_3_]^+^, 329.0662 [M + H-2CH_3_]^+^, 315.0865, 298.0840 [M + H-2CH_3_-CH_3_O]^+^, 283.0607 [M + H-3CH_3_-CH_3_O]^+^, 255.0668 [M + H-3CH_3_-CH_3_O-CO]^+^, 227.0700, 153.0168	342.0745 [M − H-CH_3_]^−^, 327.0516 [M − H-2CH_3_]^−^, 312.0262 [M − H-3CH_3_]^−^, 297.0033, 269.0098
13	Monohydroxy-tetramethoxyflavone	C_19_H_18_O_7_	16.7	359.1131 (3.3)	357.0977 (−0.8)	344.0898 [M + H-CH_3_]^+^, 329.0663 [M + H-2CH_3_]^+^, 314.0432 [M + H-3CH_3_]^+^, 311.0533, 283.0613 [M + H-3CH_3_-CH_3_O]^+^, 257.0453, 211.0240, 183.0297	342.0751 [M − H-CH_3_]^−^, 327.0513 [M − H-2CH_3_]^−^, 312.0276 [M − H-3CH_3_]^−^, 299.0565 [M − H-2CH_3_-CO]^−^, 284.0322 [M − H-3CH_3_-CO]^−^, 269.0095 [M − H-4CH_3_-CO]^−^, 207.0301, 192.0063, 117.0354
14	Monohydroxy-tetramethoxyflavone	C_19_H_18_O_7_	17.9	359.1128 (1.2)	ND	344.0882 [M + H-CH_3_]^+^, 329.0630 [M + H-2CH_3_]^+^, 311.0548, 283.0524 [M + H-3CH_3_-CH_3_O]^+^, 261.0190	ND
15	Tetramethoxyflavone*-O-*glucuronide	C_25_H_26_O_13_	11.7	535.1423 (2.7)	533.1281 (−3.6)	359.1123 [M + H-GlcUA]^+^, 344.1085 [M + H-GlcUA-CH_3_]^+^, 329.0631 [M + H-GlcUA-2CH_3_]^+^, 289.0655	ND
16	Tetramethoxyflavone*-O-*glucuronide	C_25_H_26_O_13_	12.3	535.1434 (2.9)	533.1299 (−0.6)	359.1135 [M + H-GlcUA]^+^, 344.0922 [M + H-GlcUA-CH_3_]^+^, 315.0886, 298.0884 [M + H-GlcUA-2CH_3_-CH_3_O]^+^	ND
17	Tetramethoxyflavone*-O-*glucuronide	C_25_H_26_O_13_	12.6	535.1476 (3.7)	533.1297 (−0.7)	359.1145 [M + H-GlcUA]^+^, 344.0901 [M + H-GlcUA-CH_3_]^+^, 329.0674 [M + H-GlcUA-2CH_3_]^+^, 311.0534, 298.0843 [M + H-GlcUA-2CH_3_-CH_3_O]^+^, 283.0608 [M + H-GlcUA-3CH_3_-CH_3_O]^+^	ND
18	Tetramethoxyflavone*-O-*sulfate	C_19_H_18_O_10_S	12.7	439.0706 (2.8)	437.0540 (−1.3)	359.1128 [M + H-SO_3_]^+^, 344.0889 [M + H-SO_3_-CH_3_]^+^, 329.0664 [M + H-SO_3_-2CH_3_]^+^, 271.0532, 153.0228	357.0995 [M − H-SO_3_]^−^, 342.0725 [M − H-SO_3_-CH_3_]^−^, 327.0507
19	Tetramethoxyflavone*-O-*sulfate	C_19_H_18_O_10_S	14.7	439.0698 (1.9)	437.0543 (−1.1)	359.1133 [M + H-SO_3_]^+^, 344.0938 [M + H-SO_3_-CH_3_]^+^, 329.0673 [M + H-SO_3_-2CH_3_]^+^, 311.0511, 283.0590 [M + H-SO_3_-3CH_3_-CH_3_O]^+^, 257.0479	357.0990 [M − H-SO_3_]^−^, 327.0496 [M − H-SO_3_-2CH_3_]^−^, 312.0270, 269.0075
20	Dihydroxy-tetramethoxyflavone	C_19_H_18_O_8_	14.0	375.1074 (1.6)	373.032 (−0.9)	360.0832 [M + H-CH_3_]^+^, 345.0588 [M + H-2CH_3_]^+^, 327.0467 [M + H-2CH_3_-H_2_O]^+^, 314.0813 [M + H-2CH_3_-CH_3_O]^+^, 299.0500 [M + H-3CH_3_-CH_3_O]^+^, 271.0566 [M + H-3CH_3_-CH_3_O-CO]^+^	ND
21	Dihydroxy-tetramethoxyflavone	C_19_H_18_O_8_	14.5	375.1090 (1.3)	373.0917 (−3.2)	360.0861 [M + H-CH_3_]^+^, 345.0598 [M + H-2CH_3_]^+^, 327.0496 [M + H-2CH_3_-H_2_O]^+^, 197.0062	358.0721 [M − H-CH_3_]^−^, 343.0473 [M − H-2CH_3_]^−^
22	Dihydroxy-tetramethoxyflavone	C_19_H_18_O_8_	15.1	375.1080 (3.1)	373.0924 (−0.9)	360.0829 [M + H-CH_3_]^+^, 345.0612 [M + H-2CH_3_]^+^, 327.0456, 273.0403, 256.0380	358.0720 [M − H-CH_3_]^−^, 343.0452 [M − H-CH_3_]^−^, 327.1716 [M − H-CH_3_-CH_3_O]^−^, 305.1931, 285.0019, 263.0357
23	Dihydroxy-tetramethoxyflavone	C_19_H_18_O_8_	19.4	375.1095 (2.3)	373.0919 (−2.8)	360.0828 [M + H-CH_3_]^+^, 345.0595 [M + H-2CH_3_]^+^, 327.0488 [M + H-2CH_3_-H_2_O]^+^, 313.0704, 197.0084	358.0680 [M − H-CH_3_]^−^, 343.0460 [M − H-2CH_3_]^−^, 328.0226 [M − H-3CH_3_]^−^, 312.9910
24	Monohydroxy-tetramethoxyflavone*-O-*glucuronide	C_25_H_26_O_14_	11.8	551.1396 (2.6)	ND	375.1088 [M + H-GlcUA]^+^, 360.0890 [M + H-GlcUA-CH_3_]^+^, 345.0622 [M + H-GlcUA-2CH_3_]^+^, 327.0489 [M + H-GlcUA-2CH_3_-H_2_O]^+^, 305.1602 [M + H-GlcUA-4CH_3_]^+^, 133.0879	ND
25	Nobiletin^b^ ^,^ ^c^	C_21_H_22_O_8_	19.3	403.1363 (0.5)	ND	388.1176 [M + H-CH_3_]^+^, 373.0916 [M + H-2CH_3_]^+^, 358.0858 [M + H-3CH_3_]^+^, 327.0858	ND
26	Monohydroxy-pentamethoxyflavone	C_20_H_20_O_8_	15.8	389.1255 (3.6)	387.1081 (−1.2)	374.1016 [M + H-CH_3_]^+^, 359.0770 [M + H-2CH_3_]^+^, 341.0665 [M + H-2CH_3_-H_2_O]^+^, 331.0835 [M + H-2CH_3_-CO]^+^, 316.0585 [M + H-3CH_3_-CO]^+^, 285.0766 [M + H-3CH_3_-CO-CH_3_O]^+^	372.0849 [M − H-CH_3_]^−^, 357.0620 [M − H-2CH_3_]^−^, 342.0370 [M − H-3CH_3_]^−^, 299.0162, 271.0243
27	Monohydroxy-pentamethoxyflavone	C_20_H_20_O_8_	16.5	389.1247 (2.7)	ND	374.1012 [M + H-CH_3_]^+^, 359.0777 [M + H-2CH_3_]^+^, 344.0658 [M + H-3CH_3_]^+^, 341.0635 [M + H-2CH_3_-H_2_O]^+^, 331.0823 [M + H-2CH_3_-CO]^+^, 316.0596 [M + H-3CH_3_-CO]^+^	ND
28	Monohydroxy-pentamethoxyflavone	C_20_H_20_O_8_	17.1	389.1242 (3.2)	387.1082 (−0.8)	374.1015 [M + H-CH_3_]^+^, 359.0771 [M + H-2CH_3_]^+^, 344.0542 [M + H-3CH_3_]^+^, 341.0665 [M + H-2CH_3_-H_2_O]^+^, 313.0724 [M + H-3CH_3_-H_2_O-CO]^+^, 287.0563, 244.0372, 211.0238	372.0845 [M − H-CH_3_]^−^, 357.0607 [M − H-2CH_3_]^−^, 342.0369 [M − H-3CH_3_]^−^, 327.0136 [M − H-4CH_3_]^−^, 314.0418 [M − H-3CH_3_-CO]^−^, 299.0168 [M − H-4CH_3_-CO]^−^
29	Pentamethoxyflavone*-O-*glucuronide	C_26_H_28_O_14_	12.8	565.1574 (2.5)	563.1398 (−1.4)	389.1245 [M + H-GlcUA]^+^, 374.1042 [M + H-GlcUA-CH_3_]^+^, 359.0758 [M + H-GlcUA-2CH_3_]^+^	ND
30	Pentamethoxyflavone*-O-*glucuronide	C_26_H_28_O_14_	13.1	565.1578 (3.4)	563.1402 (−0.5)	389.1251 [M + H-GlcUA]^+^, 374.1002 [M + H-GlcUA-CH_3_]^+^, 359.0774 [M + H-GlcUA-2CH_3_]^+^, 341.0664 [M + H-GlcUA-2CH_3_-H_2_O]^+^, 313.0724 [M + H-GlcUA-2CH_3_-H_2_O-CO]^+^	387.1083 [M − H-GlcUA]^−^, 372.0851 [M − H-GlcUA-CH_3_]^−^, 357.0587 [M − H-GlcUA-2CH_3_]^−^, 342.0357 [M − H-GlcUA-3CH_3_]^−^, 175.0211, 113.0255
31	Pentamethoxyflavone*-O-*sulfate	C_20_H_20_O_11_S	14.2	469.0743 (1.6)	467.0650 (−0.7)	389.1221 [M + H-SO_3_]^+^, 374.1044 [M + H-SO_3_-CH_3_]^+^, 359.0794 [M + H-SO_3_-2CH_3_]^+^, 341.0606 [M + H-SO_3_-2CH_3_-H_2_O]^+^	387.1066 [M − H-SO_3_]^−^, 357.0605 [M − H-SO_3_-2CH_3_]^−^, 342.0430 [M − H-SO_3_-3CH_3_]^−^
32	Pentamethoxyflavone*-O-*sulfate	C_20_H_20_O_11_S	14.5	469.0788 (1.2)	467.0650 (−1.8)	389.1226 [M + H-SO_3_]^+^, 374.1020 [M + H-SO_3_-CH_3_]^+^, 359.0764 [M + H-SO_3_-2CH_3_]^+^, 341.0676	387.1111 [M − H-SO_3_]^−^, 372.0868 [M − H-SO_3_-CH_3_]^−^, 357.0680, 342.0405 [M − H-SO_3_-2CH_3_]^−^, 327.0110
33	Pentamethoxyflavone*-O-*sulfate	C_20_H_20_O_11_S	14.8	469.0814 (2.2)	467.0646 (−1.5)	389.1230 [M + H-SO_3_]^+^, 374.1010 [M + H-SO_3_-CH_3_]^+^, 359.0785 [M + H-SO_3_-2CH_3_]^+^, 345.0986, 285.0794	387.1094 [M − H-SO_3_]^−^, 372.0843 [M − H-SO_3_-CH_3_]^−^, 357.0602 [M − H-SO_3_-2CH_3_]^−^, 342.0352 [M − H-SO_3_-3CH_3_]^−^, 299.0160, 264.9846, 207.0359
34	Monohydroxy-hexamethoxyflavone	C_21_H_22_O_9_	16.5	419.1342 (0.9)	417.1177 (−2.2)	404.1117 [M + H-CH_3_]^+^, 389.0872 [M + H-2CH_3_]^+^, 374.3168 [M + H-3CH_3_]^+^, 371.0768 [M + H-2CH_3_-H_2_O]^+^, 343.0765	402.1001 [M − H-CH_3_]^−^, 387.0742 [M − H-2CH_3_]^−^, 372.0433 [M − H-3CH_3_]^−^, 355.2913, 329.0341
35	Monohydroxy-hexamethoxyflavone	C_21_H_22_O_9_	17.1	419.1323 (−0.9)	ND	404.1052 [M + H-CH_3_]^+^, 389.0895 [M + H-2CH_3_]^+^, 371.0796 [M + H-2CH_3_-H_2_O]^+^, 346.0798, 328.0616	ND
36	Monohydroxy-hexamethoxyflavone	C_21_H_22_O_9_	17.8	419.1344 (1.4)	417.1176 (−3.7)	404.1093 [M + H-CH_3_]^+^, 389.0879 [M + H-2CH_3_]^+^, 371.0762 [M + H-2CH_3_-H_2_O]^+^, 346.0686, 311.0574 [M + H-6CH_3_-H_2_O]^+^, 275.0553, 211.0209, 183.0255, 151.0387	402.1048 [M − H-CH_3_]^−^, 387.0712 [M − H-2CH_3_]^−^, 371.3115, 355.2848, 349.2049, 329.0251
37	Hexamethoxyflavone*-O-*glucuronide	C_27_H_30_O_15_	13.2	595.1672 (2.4)	ND	419.1361 [M + H-GlcUA]^+^, 389.0896 [M + H-GlcUA-3CH_3_]^+^, 287.0785	ND
	Flavanone derivates						
38	Homoeriodictyol	C_16_H_14_O_6_	12.2	303.0866 (1.5)	ND	285.0755 [M + H-H_2_O]^+^, 177.0536, 153.0190 [M + H-C_9_H_10_O_2_]^+^, 117.0324	ND
39	Hesperidin^b^ ^,^ ^c^	C_28_H_34_O_15_	11.4	611.2039 (3.3)	ND	566.4279, 465.1412, 449.1442, 413.1224, 345.0988 [M + H-Rha-C_4_H_8_O_4_]^+^, 303.0872 [M + H-Rha-Glc]^+^, 285.0762 [M + H-Rha-Glc-H_2_O]^+^, 263.0545, 153.0147 [M + H-Rha-Glc-C_9_H_10_O_2_]^+^	ND
40	Hesperetin^b^ ^,^ ^c^	C_16_H_14_O_6_	16.0	303.0869 (2.3)	301.0715 (−0.8)	285.0737 [M + H-H_2_O]^+^, 177.0552, 153.0186 [M + H-C_9_H_10_O_2_]^+^, 117.0333, 89.0401	286.0488 [M − CH_3_]^−^, 242.0557 [M − H-CH_2_O-HCO]^−^, 199.0418, 164.0120 [M − H-C_8_H_9_O_2_]^−^, 151.0060 [M − H-C_9_H_10_O_2_]^−^, 136.0193, 125.0260, 108.0246
41	Hesperetin-*O*-glucuronide/Homoeriodictyol-*O*-glucuronide	C_22_H_22_O_12_	12.2	479.1190 (2.7)	477.1026 (−2.6)	461.1046 [M + H-H_2_O]^+^, 303.0892 [M + H-GlcUA]^+^, 285.0768 [M + H-GlcUA-H_2_O]^+^, 231.0244, 177.0540, 153.0180 [M + H-GlcUA-C_9_H_10_O_2_]^+^	301.0712 [M − H-GlcUA]^−^, 286.0520 [M − H-GlcUA-CH_3_]^−^, 227.0294, 175.0213, 113.0242
42	Hesperetin-*O*-sulfate/Homoeriodictyol-*O*-sulfate	C_16_H_14_O_9_S	13.0	383.0463 (1.6)	ND	303.0903 [M + H-SO_3_]^+^, 153.0204 [M + H-SO_3_-C_9_H_10_O_2_]^+^	ND
43	Naringenin^b^ ^,^ ^c^	C_15_H_12_O_5_	15.4	273.0771 (2.2)	271.0613 (0.3)	153.0186 [M + H-C_8_H_8_O]^+^, 119.0492, 91.0606	151.0043 [M − H-C_8_H_8_O]^−^, 119.0508 [M − H-C_7_H_4_O_4_]^−^, 107.0154 [M − H-C_8_H_8_O-CO_2_]^−^, 93.0381 [M − H-C_9_H_6_O_4_]^−^
44	Isosakuranetin^c^	C_16_H_14_O_5_	19.1	287.0928 (2.4)	285.0767 (−0.6)	246.8594, 167.0341 [M + H-C_8_H_8_O]^+^, 147.0431, 119.0496, 91.05551	270.0544 [M − CH_3_]^−^, 243.0657, 199.0748, 165.0130 [M − H-C_8_H_8_O]^−^, 136.0137, 119.0498
	Flavone derivates						
45	Luteolin	C_15_H_10_O_6_	14.4	287.0547 (−4.4)	ND	153.0169 [M + H-C_8_H_6_O_2_]^+^	ND
46	Apigenin^b^ ^,^ ^c^	C_15_H_10_O_5_	15.5	271.0606 (2.5)	269.0457 (0.8)	253.0497 [M + H-H_2_O]^+^, 243.0659 [M + H-CO]^+^, 215.0708, 197.0603, 153.0183 [M + H-C_8_H_8_O]^+^, 115.0541, 91.0563	241.0494 [M − H-CO]^−^, 224.0491, 201.0560, 159.0450, 133.0289, 107.0151
47	Apigenin*-O-*glucuronide	C_21_H_18_O_11_	8.3	447.0879 (0.6)	ND	350.1642, 271.0582 [M + H-GlcUA]^+^, 215.0660, 153.0222 [M + H-GlcUA-C_8_H_8_O]^+^	ND
48	Apigenin*-O-*glucuronide	C_21_H_18_O_11_	10.8	447.0930 (1.4)	445.0766 (−2.3)	ND	269.0459 [M − H-GlcUA]^−^, 113.0269
49	Apigenin*-O-*Sulfate	C_15_H_10_O_8_S	13.0	351.0182 (3.6)	349.0026 (−0.6)	271.0593 [M + H-SO_3_]^+^, 253.0471 [M + H-SO_3_-H_2_O]^+^, 243.0619 [M + H-SO_3_-CO]^+^, 215.0704, 153.0175 [M + H-SO_3_-C_8_H_8_O]^+^	269.0457 [M − H-SO_3_]^−^, 241.0487, 225.0567, 213.0518, 151.0048 [M − H-SO_3_-C_8_H_8_O]^−^, 117.0345 [M − H-SO_3_-C_7_H_4_O_4_]^−^

a The losses are: Glc, glucose moiety; Rha, rhamnose moiety; ND, not detect.

b Confirmation in comparison with authentic standards.

c Confirmation in comparison with mass spectral library (Natural Products HR-MS/MS Spectral Library, Version 1.0; AB Sciex, Foster City, United States).

Nobiletin is a hexamethoxyflavone abundant in CRP and has been documented to possess multiple pharmacological activities, such as anti-inflammatory, bacteriostasis and antioxidant (Li et al., 2014). As shown in Table 2, nobiletin gave its quasi-molecular ion [M + H]^+^ at *m/z* 403.1363, yielding the characteristic ions at *m/z* 388.1176, 373.0916, 358.0858 with successive loss of CH_3_. Compound 26, 27, 28 all possessed the [M + H]^+^ ion at *m/z* 389, which was is 14 Da (CH_2_) less than protonated nobiletin. Herein compound 26, 27, 28 were tentatively assigned as the mono-demethylated metabolite of nobiletin. Using *in vitro* incubation methods, Koga et al. (Koga et al., 2007) investigated the metabolism of nobiletin in rat liver microsomes. As a result, three mono-demethylated metabolites were identified, which were 4′-OH-, 7-OH-, and 6-OH-nobiletin. Based on the retention time, compound 26, 27, 28 were identified as 6-OH-, 7-OH-, and 4′-OH-nobiletin, respectively. These metabolites could be subsequently demethylated into several dihydroxy-tetramethoxyflavone, while the position of the hydroxyl group needs to be further assigned. These hydroxyl-containing polymethoxyflavones could be further combined with glucuronic acid or sulfuric acid to generate corresponding phase II metabolites.

**FIGURE 6:**
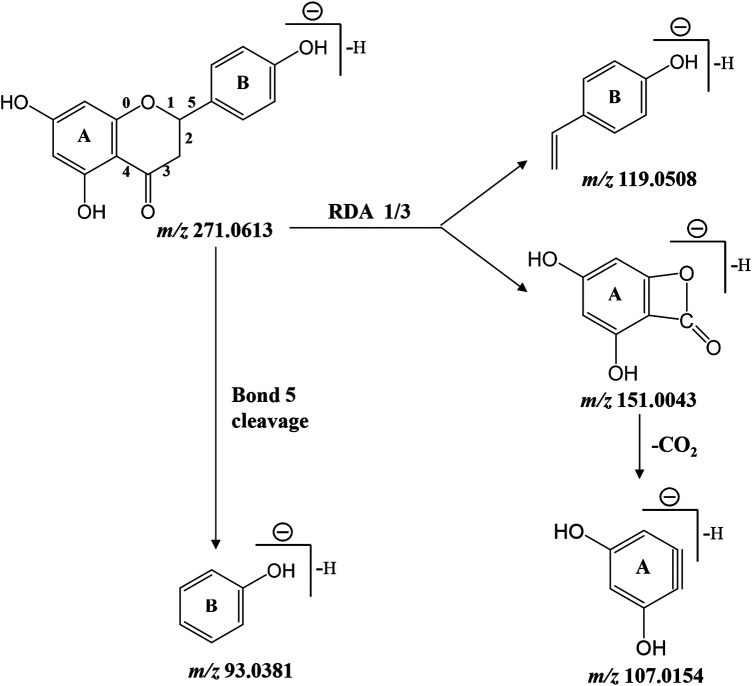
Proposed fragmentation pattern of deprotonated naringenin.

As presented in Table 2, retro Diels-Alder reaction was a characteristic pattern of flavanone and flavone derivates in MS/MS fragmentation (Zeng et al., 2020b). Taking naringenin as an example, in the negative ion mode, the main fragment ions of deprotonated naringenin (*m/z* 271.0613) were *m/z* 151.0043, 119.0508, 107.0154, and 93.0381. Among them, product ions *m/z* 151.0043 (^1, 3^A^−^) and 119.0508 (^1, 3^B^−^) were presumed to be produced by retro Diels-Alder reaction on chemical bond 1 and 3, while *m/z* 107.0154 was derived from ^1, 3^A^−^ with the loss of CO_2_ (44 Da). The signal at *m/z* 93.0381 was presumed to be yielded by the breaking of chemical bond 5. Above fragmentation schemes were proposed in Figure 6.

Oral ingestion is the most common mode of administration used in herbal medicine. Generally speaking, exogenous compounds could only exert its pharmacological activity if it is absorbed into the blood and maintains a certain concentration. Urine is filtered from the blood and accumulated all changes in the body. It contains all metabolites in the blood, and thus comprehensively reflects the metabolic information. In this work, the concentration of prototype compounds detected in urine was much lower than that of the corresponding metabolites, suggesting that flavonoid metabolites may be the primary substance for pharmacological activity of CRP, rather than its prototype compounds. Therefore, identified metabolites were employed in subsequent network pharmacology analysis so as to discover the bioactive components and potential targets of CRP against spleen deficiency.

### Network Pharmacology Analysis

**FIGURE 7:**
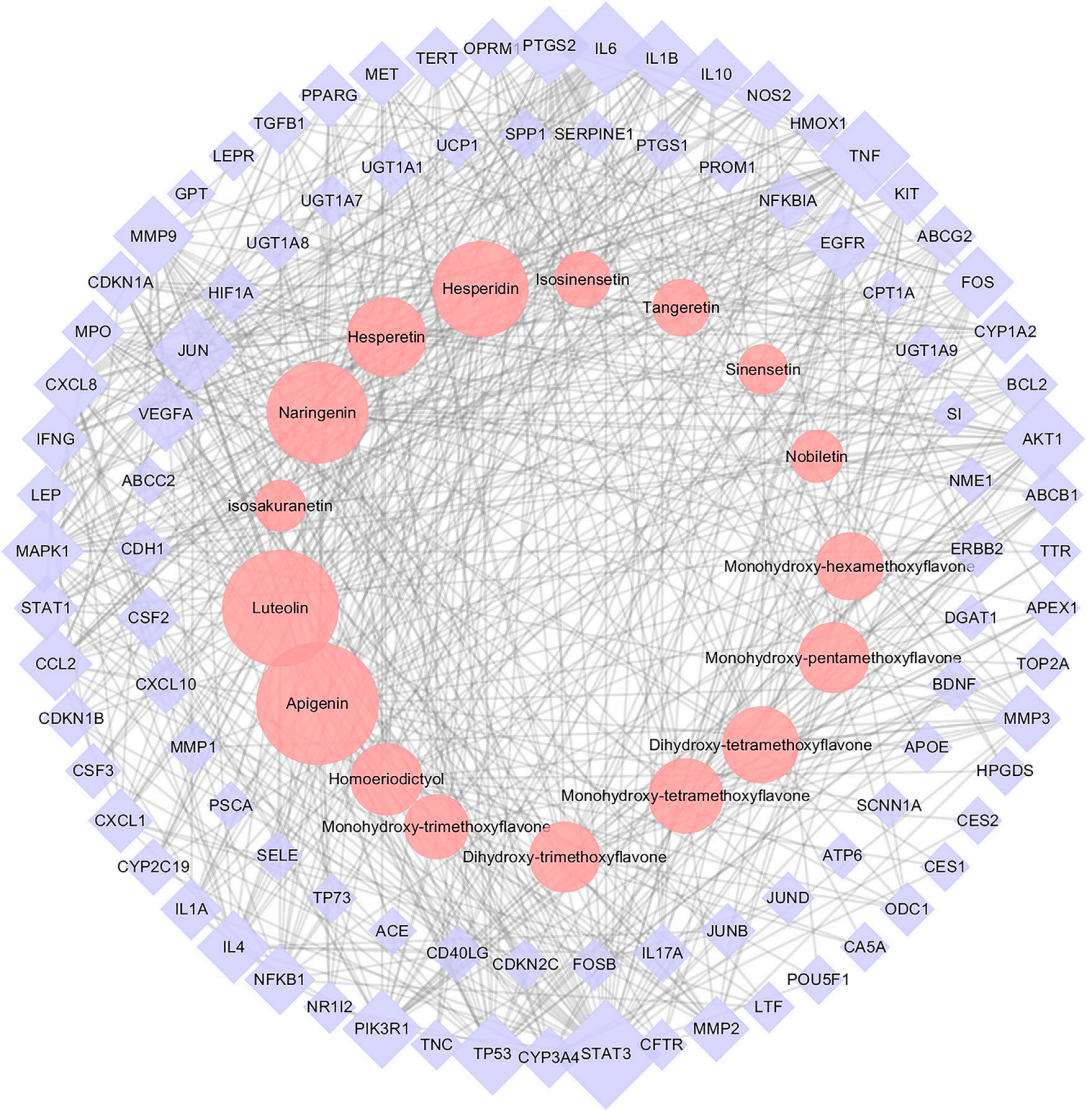
The Citri Reticulatae Pericarpium (CRP)-related target-spleen deficiency target network (CT-ST network). Pink circles, purple diamonds represented CRP compounds and the common targets for both compounds and spleen deficiency, respectively. The size of circles and diamonds indicated nodes degree value.

On the basis of identified metabolites, a total of 583 potential targets for CRP’s efficacy were defined. Meanwhile, a total of 520 spleen deficiency-related disease targets were acquired by integrating data on diarrhea, dyspepsia, enteritis and gastritis from disease databases. Detailed information was presented in Supplementary Table S1. The CRP’s efficacy-related targets were mapped to the spleen deficiency-related targets, and as a result, 98 common targets were obtained. Then a PPI network (Supplementary Figure S1) was constructed using the common targets and further used to construct the CT-ST network. Figure 7 illustrated the CT-ST network which consists of 111 nodes (17 compounds and 94 candidate targets, compounds with multiple isomers are combined and shared one name) and 662 edges. Among them, several flavonoids including apigenin, luteolin, naringenin, hesperidin, hesperetin, dihydroxy-tetramethoxyflavone, monohydroxy- tetramethoxyflavone and homoeriodictyol were considered to be key compounds for CRP to alleviate spleen deficiency. The primary targets of these compounds included STAT3, IL6, TNF, JUN, AKT1, TP53, MAPK1, MMP9, PIK3R1, PTGS2, VEGFA, EGFR, IL1B, CXCL8, IL4, CCL2, IL10, and FOS. That is to say, CRP may interact with these targets to exert its effects in relieving spleen deficiency.

**FIGURE 8:**
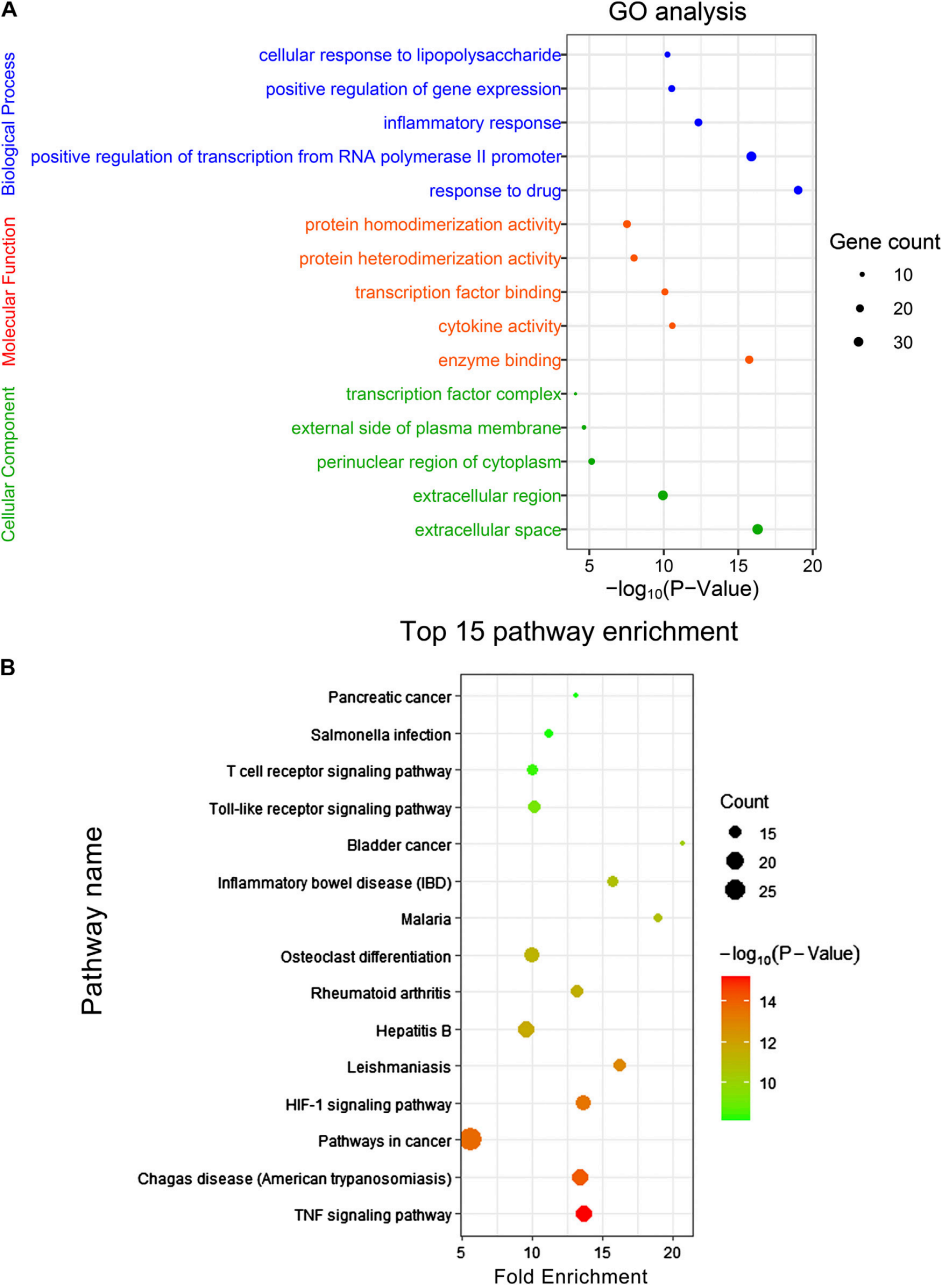
**(A)** GO analysis and **(B)** KEEG signaling pathway enrichment of the common targets. The size of the dot indicates the number of target genes in the pathway and the color of the dot reflects the *p* values.

In order to further reveal the molecular mechanism of CRP against spleen deficiency, GO analysis and pathway enrichment of mentioned 94 common targets were performed with KEGG database. The results of GO analysis were described by biological process (BP), cell component (CC), and molecular function (MF) terms. In GO analysis, 330 of 426 BPs, 58 of 72 MFs, and 26 of 33 CCs enriched for these targets were recognized as *p* < 0.05. Top 5 enriched terms of BP, MF, CC categories in the GO analysis were presented in Figure 8A. Moreover, 97 target-related pathways were found in KEGG, top 15 KEEG signaling pathways were constructed in bubble plot based on *P*-Value (Figure 8B), and the involved genes were showed in Supplementary Table S2. Consistent with the pharmacological activities, network pharmacology analysis revealed that the interaction between CRP and spleen deficiency involved multiple BPs and pathways, including inflammatory responses, immune system, and oxidative stress, mediating by TNF signaling pathway, hypoxia-inducible factor-1 (HIF-1) signaling pathway, Toll-like receptor signaling pathway, *etc*. Reported studies showed that pro-inflammatory and/or oxidative stress mediators are directly interlinked with the pathogenesis of many gastrointestinal diseases (Chung et al., 2016). Among mentioned pathways, TNF signaling is critical to the maintenance of intestinal barrier and epithelial cell tight junctions (Kolodziej et al., 2011). HIF-1 signaling pathway is closely related to stress-responsive gene expression (Surazynski et al., 2008). Toll-like receptor signaling pathway is important for maintaining tolerance to commensal microbiota and inducing inflammation against pathogens, hence playing an essential role in homeostasis of the intestine (Biswas et al., 2011; Kamdar et al., 2013). However, further mechanism studies are necessary to assign the role of these pathway in CRP against spleen deficiency.

As shown in Figure 7, a total of 17 compounds (mainly flavonoids) were assigned as the core bioactive components in CRP by network pharmacology analysis. Our preliminary chemical profile analysis suggested that these compounds were abundant in CRP herbs (Zheng et al., 2019). Besides mentioned targets and pathways, these compounds could also interact with gut microbiota after oral administration, which probably contribute to the overall therapeutic effects of CRP against spleen deficiency. Take hesperetin as an example, ingestion of an assigned diet (0.5% hesperetin) for three weeks could significantly affect the structure and activity of gut microbiota in rats (Unno et al., 2015). The richness of *Clostridium subcluster XIVa* in feces was significantly reduced, while the cecal SCFA pool was noticeably increased. Therefore, in the follow-up study to clarify the mechanism of action, we should not only pay attention to identified targets and pathways but also focus on gut microbiota.

## Conclusion

In this study, the efficacy against spleen deficiency and gut microbiota modulatory properties of CRP were investigated using reserpine treated rats as the animal model. CRP derived metabolites were identified in rat urine and further applied to explore the core bioactive components and potential targets through network pharmacology analysis. As a result, CRP administration could effectively alleviate the typical symptoms of spleen deficiency induced by reserpine treatment, including poor digestion and absorption capacity, and disorder in gastrointestinal hormones, immune cytokines and oxidative stress. Meanwhile, CRP was found to improve the diversity and structure of the gut microbiota in spleen deficiency rats. Compared with model group, some SCFAs producing and anti-inflammatory bacteria including *Bifidobacterium*, *Lactobacillus*, *Allobaculum*, *Psychrobacter*, *[Eubacterium]_ coprostanoligenes_group*, *Prevotellaceae_Ga6A1_group*, *Parasutterella* were up-regulated, while some spleen deficiency aggravated related bacteria including *Alistipes*, *Anaerotruncus*, *Desulfovibrio*, *Oscillibacter*, *Ruminiclostridium_9*, *Ruminococcaceae_UCG-003*, *Ruminiclostridium_5*, *Parabacteroides* were down-regulated in CRP group rats. Further Spearman's correlation analysis indicated that there existed a close correlation between the profiles of gut microbiota and the spleen-deficiency related biochemical indexes. In addition, a total of 26 prototype compounds and 23 metabolites were detected in rat urine after the ingestion of CRP. Through network pharmacology analysis, apigenin, luteolin, naringenin, hesperidin, hesperetin, dihydroxy-tetramethoxyflavone, monohydroxy-tetramethoxyflavone and homoeriodictyol were assigned as the core bioactive components, while STAT3, IL6, TNF, JUN, AKT1, TP53, MAPK1, MMP9, PIK3R1, PTGS2, VEGFA, EGFR, IL1B, CXCL8, IL4, CCL2, IL10, and FOS were defined as the major potential targets. Further GO analysis and pathway enrichment suggested that therapeutic effects of CRP against spleen deficiency involved multiple BPs, including inflammatory responses, immune system and oxidative stress such as TNF signaling pathway, HIF-1 signaling pathway, and Toll-like receptor signaling pathway. Besides these targets and pathways, identified compounds could also interact with gut microbiota after oral administration, which probably contribute to the overall therapeutic effects of CRP. This work provided systematic insights to understand the mechanism of CRP alleviating spleen deficiency-related diseases.

## Data Availability Statement

The datasets generated for this study can be found in GenBank accession numbers (MW015148–MW015742).

## Ethics Statement

The animal study was reviewed and approved by Animal Ethics Committee of the School of Life Sciences in Sun Yat-sen University.

## Author Contributions

YZ, WP, and WS conceived and designed the study. YZ, XZ, and TC carried out the experiments. YZ, XZ, and PC analyzed the research data and wrote the manuscript.

## Funding

This research was supported by the Science and Technology Planning Project of Guangdong Province in China (2019B090905002).

## Conflict of Interest

The authors declare that the research was conducted in the absence of any commercial or financial relationships that could be construed as a potential conflict of interest.
