# Supplementary figures and images for: Integrating Pharmacology and Gut Microbiota Analysis to Explore the Mechanism of Citri Reticulatae Pericarpium Against Reserpine-Induced Spleen Deficiency in Rats
Source: Front Pharmacol. 2020 Oct 20;11:586350. doi: 10.3389/fphar.2020.586350 (PMC7606944; doi:10.3389/fphar.2020.586350)

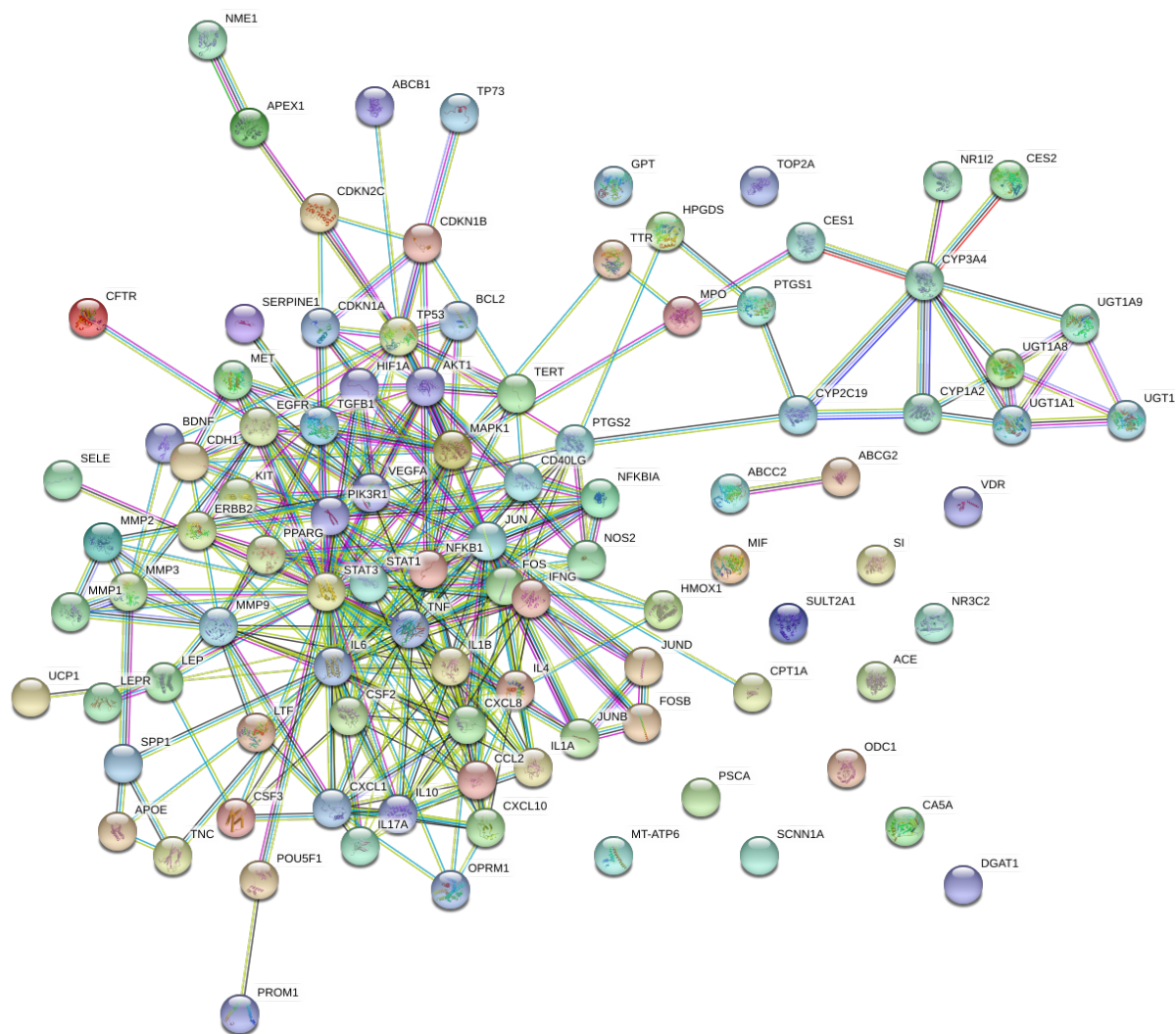

Figure S1. The protein protein interaction (PPI) network.

Supplement: Supplementary file 1 [file Image1_v1.pdf]
