# Supplementary material for: Integrating Pharmacology and Gut Microbiota Analysis to Explore the Mechanism of Citri Reticulatae Pericarpium Against Reserpine-Induced Spleen Deficiency in Rats
Source: Front Pharmacol. 2020 Oct 20;11:586350. doi: 10.3389/fphar.2020.586350 (PMC7606944; doi:10.3389/fphar.2020.586350)
Supplement: Supplementary file 3 [file Table2_v2.docx]

Table S2 Genes involved in the top 15 KEGG enriched pathways

| No. | Pathway | Percent | Gene name |
| --- | --- | --- | --- |
| 1 | TNF signaling pathway | 20.2 | CXCL1, CSF2, IL6, TNF, CCL2, PTGS2, MMP9, NFKBIA, NFKB1, MMP3, JUNB, CXCL10, AKT1, MAPK1, FOS, JUN, IL1B, SELE, PIK3R1 |
| 2 | Chagas disease (American trypanosomiasis) | 19.1 | IL6, TNF, CCL2, CXCL8, NFKBIA, NFKB1, IL10, TGFB1, AKT1, MAPK1, FOS, ACE, JUN, IFNG, SERPINE1, IL1B, NOS2, PIK3R1 |
| 3 | Pathways in cancer | 30.9 | PTGS2, ERBB2, MMP9, PPARG, CXCL8, NFKBIA, NFKB1, CDH1, KIT, MMP2, TGFB1, MMP1, AKT1, FOS, BCL2, NOS2, PIK3R1, EGFR, IL6, MET, TP53, STAT1, STAT3, MAPK1, CDKN1A, CDKN1B, HIF1A, JUN, VEGFA |
| 4 | HIF-1 signaling pathway | 18.1 | EGFR, IL6, ERBB2, NFKB1, STAT3, AKT1, MAPK1, CDKN1A, HIF1A, CDKN1B, BCL2, HMOX1, IFNG, SERPINE1, VEGFA, NOS2, PIK3R1 |
| 5 | Leishmaniasis | 16 | IL4, TNF, PTGS2, NFKBIA, NFKB1, STAT1, IL10, TGFB1, MAPK1, FOS, JUN, IFNG, IL1B, NOS2, IL1A |
| 6 | Hepatitis B | 19.1 | IL6, TNF, MMP9, TP53, CXCL8, NFKBIA, NFKB1, STAT1, STAT3, TGFB1, AKT1, MAPK1, FOS, CDKN1A, CDKN1B, JUN, BCL2, PIK3R1 |
| 7 | Rheumatoid arthritis | 16 | CSF2, IL6, TNF, CCL2, CXCL8, MMP3, TGFB1, MMP1, FOS, IL17A, JUN, IFNG, VEGFA, IL1B, IL1A |
| 8 | Osteoclast differentiation | 18.1 | TNF, PPARG, NFKBIA, NFKB1, FOSB, STAT1, TGFB1, JUNB, AKT1, MAPK1, FOS, JUN, IFNG, JUND, IL1B, PIK3R1, IL1A |
| 9 | Malaria | 12.8 | CSF3, IL6, TNF, CCL2, CD40LG, MET, IFNG, CXCL8, IL1B, SELE, TGFB1, IL10 |
| 10 | Inflammatory bowel disease (IBD) | 13.8 | IL4, IL6, IL17A, TNF, JUN, IFNG, IL1B, NFKB1, STAT1, IL10, TGFB1, IL1A, STAT3 |
| 11 | Bladder cancer | 11.7 | EGFR, MAPK1, CDKN1A, MMP9, ERBB2, VEGFA, TP53, CXCL8, CDH1, MMP2, MMP1 |
| 12 | Toll-like receptor signaling pathway | 14.9 | IL6, TNF, NFKBIA, CXCL8, NFKB1, STAT1, CXCL10, AKT1, FOS, MAPK1, JUN, IL1B, PIK3R1, SPP1 |
| 13 | T cell receptor signaling pathway | 13.8 | IL4, CSF2, TNF, NFKBIA, NFKB1, IL10, AKT1, FOS, MAPK1, CD40LG, JUN, IFNG, PIK3R1 |
| 14 | Salmonella infection | 12.8 | CXCL1, CSF2, MAPK1, FOS, IL6, JUN, IFNG, CXCL8, IL1B, NFKB1, NOS2, IL1A |
| 15 | Pancreatic cancer | 11.7 | AKT1, EGFR, MAPK1, ERBB2, VEGFA, TP53, NFKB1, STAT1, TGFB1, PIK3R1, STAT3 |
